# Response time differences between men and women during hand mental rotation

**DOI:** 10.1371/journal.pone.0220414

**Published:** 2019-07-26

**Authors:** Hideki Mochizuki, Kotaro Takeda, Yutaka Sato, Izumi Nagashima, Yusuke Harada, Nobuaki Shimoda

**Affiliations:** 1 Department of Occupational Therapy, Faculty of Health Sciences, Kyorin University, Tokyo, Japan; 2 Faculty of Rehabilitation, School of Healthcare, Fujita Health University, Mie, Japan; 3 Graduate School of Technology, Industrial and Social Sciences, Tokushima University, Tokushima, Japan; 4 Department of Rehabilitation, Faculty of Health Sciences, Tokyo Kasei University, Saitama, Japan; University of Ottawa, CANADA

## Abstract

This study explored gender differences in correct response rates and response times on a task involving left or right arrow selection and another involving the transformation of mental rotation of the hand. We recruited 15 healthy, right-handed men (age 24.5 ± 6.4) and 15 healthy, right-handed women (age 21.3 ± 4.9). For the tasks, we used pictures of left and right arrows and 32 hand pictures (left and right, palm and back) placed in cons (each at 45° from 0° to 315°). Hand and arrow pictures alternated and were shown at random. Participants decided as quickly as possible whether each picture was left or right. To compare the time taken for the transformation of mental rotation of the hand, we subtracted the average arrow response time from that for the left and right hand pictures for each participant. Correct response rates did not differ significantly between men and women or left and right for either arrow or hand pictures. Regardless of gender, the response time was longer for the left arrow picture than right arrow picture. The response time for the hand picture was longest for both men and women for pictures at rotation angles that were most difficult to align with participants’ hands. While there was no difference between men’s responses for left and right hand pictures, the responses of women were longer for left than right hand pictures and also than those of men. These findings suggest that both men and women mainly perform the hand mental rotation task with implicit motor imagery. On the other hand, the gender difference in performance might be explained by the difference in balance with other strategies, such as visual imagery, and by cognitive, neurophysiological, and morphological differences.

## Introduction

The figure mental rotation task (figure MRT) is typically used to investigate spatial cognition [1, 2]. This task involves presenting participants with pairs of three-dimensional (3D) figures projected in two dimensions and having them decide whether the two figures are the same (with response time (RT) as the dependent variable of interest) [3]. The figure pairs are either identical or mirror images of each other, and the second image is rotated from the original to varying degrees. Since RTs increase in proportion to the angular rotational difference between the figure pairs, it is thought that the figure MRT is completed by mentally rotating one of the figures and placing the two figures on top of each other.

The hand mental rotation task (HMRT) is a type of MRT. In the task, participants are presented with pictures or line drawings of a hand at various rotation angles and asked to decide whether a left or right hand is being shown. Results for RT and brain function measurements during this task show that participants may mentally rotate their own hand to place it on top of the displayed hand picture [4–9].

Several studies have revealed gender differences in figure MRT performance. In general, men perform better than women [10–16]. As a reason for the gender differences, Wei et al. showed that the gray matter volume of a male’s right anterior hippocampus, which acquires or encodes new visuospatial information, is larger than that of a female [17]. On the other hand, few studies have assessed gender differences in the HMRT, and findings are mixed. For example, some studies have shown that gender does not seem to affect correct response rates [18, 19], while others found that women tend to select a higher number of incorrect responses [20]. Some research has also shown that women tend to have longer RTs [20], and others that men demonstrate longer RTs than do women [18]. These conflicting findings demonstrate that gender differences in HMRT performance are not fully understood. In the present study, we aimed to clarify the gender differences in performance ability and strategy in HMRT, using correct response rate and RT as indicators. We also aimed to assess a more accurate RT for the hand mental rotation by subtracting the time of the simple motion.

## Materials and methods

### Participants

We recruited 15 healthy, right-handed men and 15 healthy, right-handed women (men: age 24.5 ± 6.4 years, height 171.8 ± 6.0 cm, body weight 62.1 ± 15.2 kg; women: age 21.3 ± 4.9 years, height 159.0 ± 6.8 cm, body weight 49.3 ± 6.8 kg, mean ± standard deviation) and obtained their written consent to participate. There was no difference in the distribution of the presence or absence of exercise habits (more than 30 minutes per day and more than twice a week, lasting more than 1 year) between men and women (presence/absence: 3/12 for men and 1/14 for women; *p* = .60, Fisher’s exact test). We determined right-handedness by calculating the laterality quotient based on the Edinburgh Handedness Inventory [21] (men: 99.1 ± 3.3; women: 100 ± 0.0). The study was carried out with the approval of the Kyorin University ethics committee.

### Experimental procedure

Participants sat at a desk in a quiet room in front of a laptop (CF-F10, Panasonic Corp., Kadoma, Japan), and were asked to place the index finger of their left hand on the F key and index finger of their right hand on the J key of the keyboard. Their hands were then covered.

**Fig 1:**
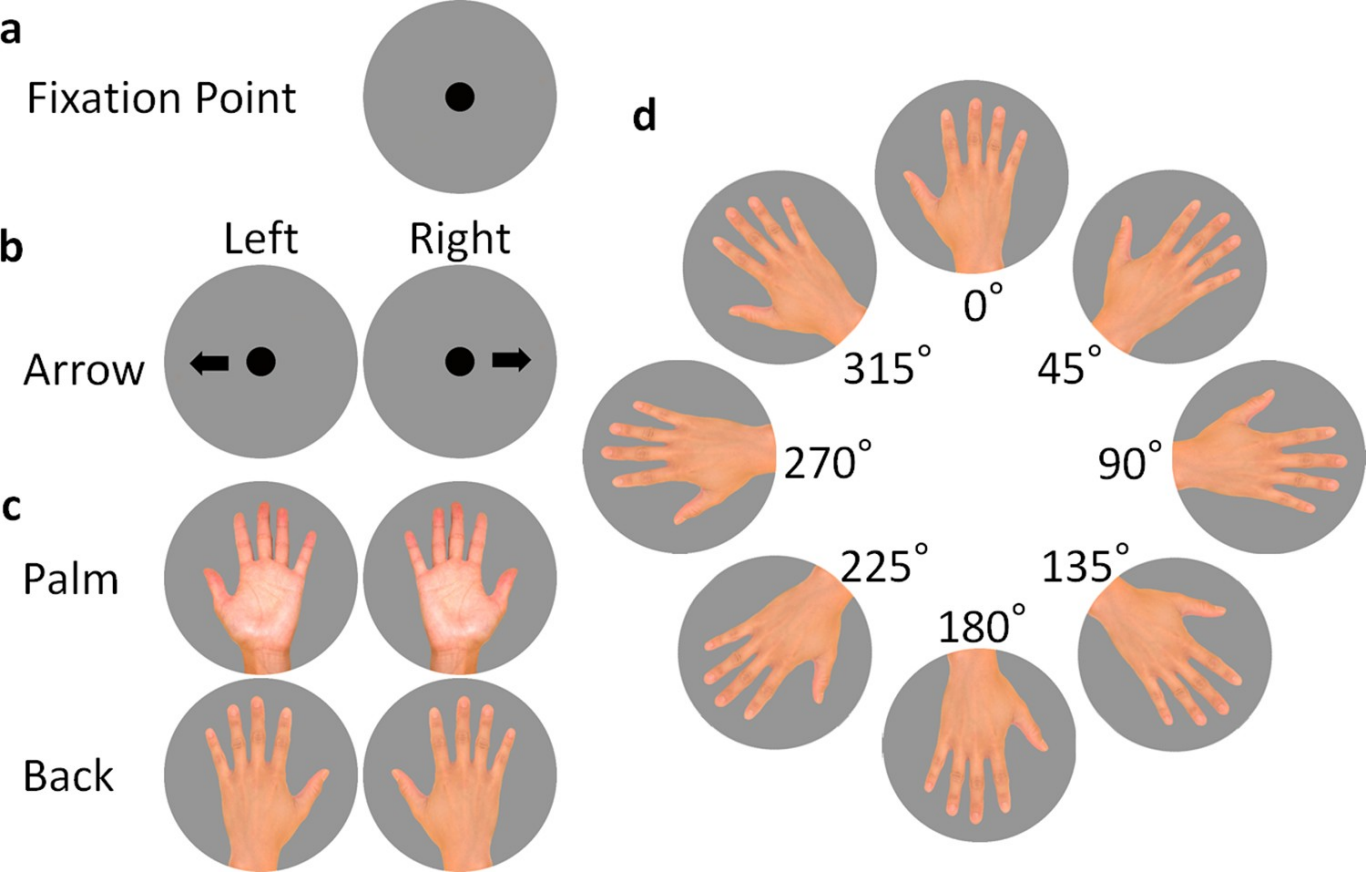
The hand and arrow pictures used in this experiment. (a) Fixation point, (b) left or right pointing arrow pictures, (c) left and right hands in palm and back view, and (d) eight rotation angles.

We used a fixation point, arrow pictures (left and right), and hand pictures (back and palm of the left and right hands), as shown in Fig 1a–1c. As shown in Fig 1d, we used pictures of the hand rotated clockwise at eight 45° angles from an original position of the third finger pointing in a vertical direction at 0 degrees (0°, 45°, 90°, 135°, 180°, 225°, 270°, and 315°).

We used image presentation and timing software (EXPLAB Ver. 1.3, Yachiyo Shuppan, Tokyo, Japan) to display the fixation point for 1 second on a 14.1-inch liquid crystal screen before presenting either a left or right arrow or hand picture. When an arrow was displayed, participants had to decide whether it was pointing to the left or right. When a hand was displayed, they had to decide whether it was a left or right hand. Participants were instructed to press the key corresponding to their decision (F for left and J for right) as quickly and accurately as possible. The arrow and hand pictures were displayed alternately, and a fixation point appeared before each presentation of the arrow or hand pictures. The fixation point was presented immediately after the participants responded, and the next image (arrow or hand) was presented after one second. There was no RT limit. The direction of the arrow (right or left) was randomly assigned compared to the laterality of the following hand picture. One set consisted of 32 arrow pictures (left or right arrow: 2 × 16 = 32) and 32 hand pictures (left or right hand × back or palm of hand × rotation angle: 2 × 2 × 8 = 32). Each participant was shown three sets at random, and we recorded the time from the picture being displayed until the key was pressed, and whether the response was correct.

A questionnaire was administered to the participants after they completed the experiment. Participants were asked to select an item closest to their performance strategy during the HMRT from the following five items: 1) mentally rotated the participant’s own hand to the presented hand picture (motor imagery), 2) mentally rotated the hand picture to an angle to judge left and right easily (visual imagery), 3) both strategies were performed, 4) not conscious of the strategy, and 5) other (please specify).

### Data analyses

Certain issues must be considered in terms of how participants carry out the HMRT. In our previous studies and most others to date [7–9, 22, 23], participants were asked to press a button with their left hand if the stimulus is a picture of a left hand, and with their right hand if the stimulus is a picture of a right hand. RTs were calculated as the length of time from being shown the picture to pressing the button. This assumes there are no differences in the time it takes to press a button (RT) after deciding which one to press. However, a study using a choice response time task (CRTT), wherein participants had to choose to respond with either their left or right hand, found that left hand RTs were longer than right hand RTs [24]. This indicates that since MRT involves visual encoding, transformation of mental rotation, comparison, decision making, and motor response generation [18], we need to consider the differences between left and right hand responses from the decision phase to the motor generation phase of the task. Zapparoli et al. [25] considered individual age-related differences in motor function and subtracted the baseline RT, which did not include a mental rotation from HMRT RT. Our previous studies [26, 27] investigated the performance strategy in the HMRT of patients with hemiplegic stroke and schizophrenia by subtracting the RTs for the arrow task from those for the HMRT (ΔRT). The present study used a CRTT with left and right arrows to investigate the differences in the RT for left and right hand responses, and between participant groups (men and women). This enabled examining gender differences in task performance and task strategies using correct response rates and RTs as indicators.

The RT for arrows was averaged for the left and right, respectively (16 pictures for each side × 3 sets), which was subtracted from the time for the hand picture of the same side. The resulting length of time (ΔRT) represents the time excluding the motor generation phase of the HMRT. For each angle and palm/back of the hand pictures, the ΔRT was averaged over three sets. These calculations were performed for each participant, excluding incorrect responses. We then compared the correct response rates, arrow picture RTs, and ΔRTs for the hand pictures.

### Statistical analyses

We performed two-way analyses of variance (ANOVAs) to analyze the correct response rates and RTs for the arrow pictures with gender (men, women) as a between-participant factor and direction (left, right) as a within-participant factor. For the hand pictures, we used three-way ANOVAs with gender (men, women) as a between-participant factor and left/right hand and rotation angle (0°, 45°, 90°, 135°, 180°, 225°, 270°, and 315°) as within-participant factors. We used Bonferroni’s method to control for multiple comparisons, and set *p* < .05 as the significance threshold. Moreover, *ε*_GG_ was calculated for the Greenhouse-Geisser correction, and the degrees of freedom were corrected when the data did not satisfy the assumption of sphericity. A Fisher’s exact test was performed to compare the distribution of the responses to the questionnaire of men and women.

SPSS Statistics software (Ver. 21.0, IBM Corporation, Armonk, USA) was used for all analyses.

## Results

Table 1 shows the correct response rates (Mean ± Standard error of the mean, *M* ± *SEM*). For the arrow pictures, no significant main effect or interactions were found for the gender and direction factors. This was also the case for the gender, left/right hand, and rotation angle factors for the hand picture ANOVA.

**Table 1:** Correct rates (%) for arrows and hand pictures (M ± SEM).

	arrows		hand pictures	
	left	right	left	right
men	99.3 ± 0.33	99.7 ± 0.19	95.0 ± 1.89	94.0 ± 1.66
women	99.4 ± 0.25	99.9 ± 0.14	92.3 ± 1.12	95.7 ± 0.94

For the arrow picture RTs, there was no significant main effect of gender (*F*(1, 28) = 0.53, *p* = .47) or interaction between gender and arrow direction (*F*(1, 28) = 0.17, *p* = .69). However, there was a main effect of arrow direction (*F*(1, 28) = 4.58, *p* < .05), with RTs for the left arrow (0.47 ± 0.01 s) significantly longer than for the right arrow (0.45 ± 0.01 s). In the analyses of the ΔRT for the hand pictures, the results of the ANOVA showed a main effect for rotation angle (*F*(7, 196) = 29.74, *ε*_GG_ = 0.50, *p* < .01), and significant interactions for left/right hand × rotation angle (*F*(7, 196) = 6.96, *ε*_GG_ = 0.77, *p* < .01) and gender × left/right hand (*F*(1, 28) = 6.42, *p* < .05). There was no significant main effect for left/right hand (*F*(1, 28) = 2.55, *p* = .12) and gender (*F*(1, 28) = 2.62, *p* = .12), or no significant interaction for gender × left/right hand × rotation angle (*F*(7, 196) = 1.11, *p* = .37) and gender × rotation angle (*F*(7, 196) = 0.78, *p* = .60).

**Fig 2:**
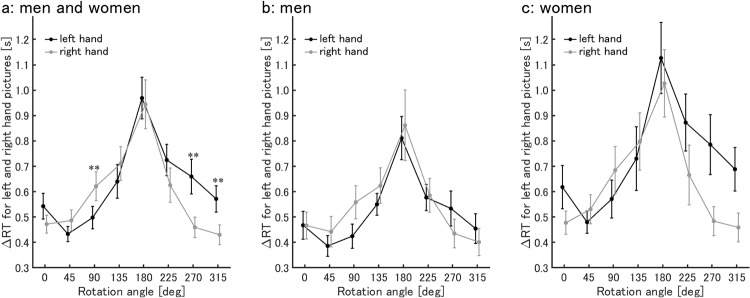
ΔRT for hand pictures in each rotation angle. ΔRT for left (black) and right (gray) hand pictures in each rotation angle (M ± SEM). (a) Both men and women, (b) men, and (c) women. **: statistical difference between left and right for the corresponding angle at *p* < .01.

Fig 2a shows the relationship between left/right hand pictures and the rotation angle. At 90°, the RTs for the right hand picture (0.62 ± 0.06 s) were significantly longer than for the left hand picture (0.50 ± 0.04 s) (Bonferroni corrected *p* < .05). At 270°, the RTs for the left hand picture (0.66 ± 0.07 s) were significantly longer than for the right hand picture (0.46 ± 0.04 s). Similarly, at 315°, the RTs for the left hand picture (0.57 ± 0.05 s) were significantly longer than for the right hand picture (0.43 ± 0.04 s). At 0°, 45°, 135°, 180°, and 225°, no significant difference was evident between the left and right hand pictures (at 0°, 0.54 ± 0.05 s vs. 0.47 ± 0.04 s, *p* = .07; at 45°, 0.43 ± 0.03 s vs. 0.49 ± 0.04 s, *p* = .11; at 135°: 0.64 ± 0.07 s vs. 0.71 ± 0.07 s, *p* = .11; at 180°, 0.97 ± 0.08 s vs. 0.94 ± 0.10 s, *p* = .68; at 225°, 0.72 ± 0.06 s vs. 0.63 ± 0.07 s, *p* = .07).

The relationships between left/right hand pictures and rotation angle for men and women are shown in Fig 2b and 2c, respectively. Multiple comparisons could not be performed, because there was no significant interaction for gender × left/right hand × rotation angle.

**Fig 3:**
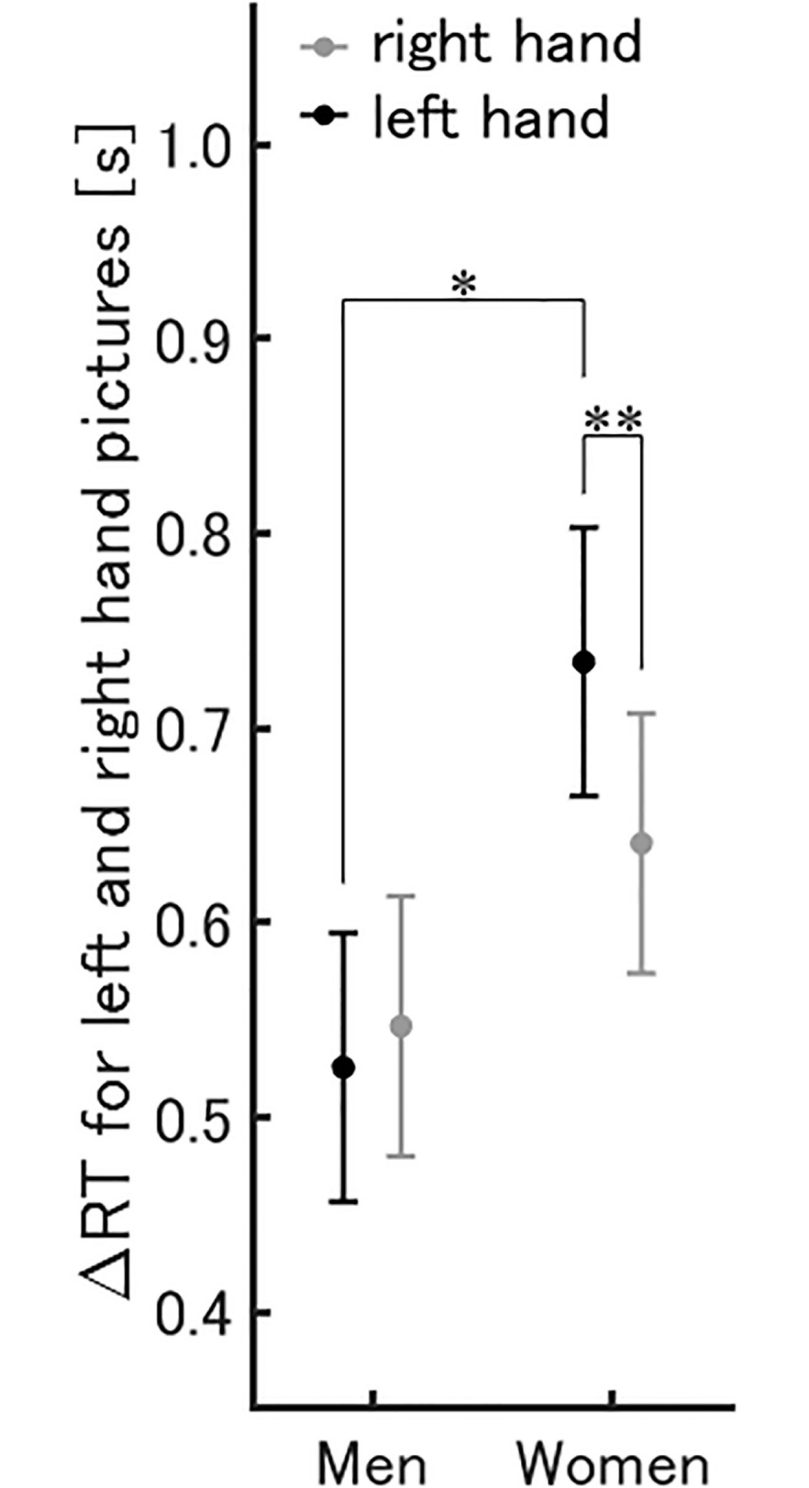
ΔRT for hand pictures in men and women. ΔRT for left (black) and right (gray) hand pictures for men and women (M ± SEM), *p < .05 and **p < .01.

Women’s ΔRT for the all left hand pictures (0.73 ± 0.07 s) was significantly longer than for the all right hand pictures (0.64 ± 0.07 s), and longer than the men’s ΔRT for all left hand pictures (0.53 ± 0.07 s). No differences between all left hand pictures (0.53 ± 0.07 s) and all right hand pictures (0.55 ± 0.07 s), *p* = .51 were identified for the male participants (Fig 3).

Table 2 shows the distribution of participants’ answers to the questionnaire. There were no significant differences in the distribution between men and women.

**Table 2:** Counted questionnaire answers.

Q. How to perform the HMRT?	Sex		*p* value
	Men	Women	
1) Motor imagery	5	7	0.80
2) Visual imagery	4	4	
3) Both strategies	3	3	
4) Not conscious	2	1	
5) Other	1*	0	

*based on the position and orientation of the thumb finger

## Discussion

This study examined whether there were differences in responses to left and right arrows in a group of 15 healthy, right-handed men and 15 healthy, right-handed women. We also investigated gender differences for the HMRT considering participants’ simple responses to the arrows.

Similar to previous findings regarding differences between left and right on a CRTT [24], our findings showed that RT was longer for the left arrow than right arrow. Although the aim of comparing the cognitive process involved in carrying out the HMRT was to compare the time needed purely for mental rotation transformation, differences between left and right motor response generation appear to have affected the time taken to press the key after the hand picture was presented. In this study, we believe we were able to accurately compare the time needed for the transformation of mental rotation of the hand, similar to previous studies [25–27]. We did this by subtracting the arrow picture RT, which did not include the cognitive processes of mental rotation transformation and comparison, from the hand picture RT. We then defined the time taken in the MRT cognitive process [18] from being presented with the hand picture to deciding whether it was left or right as the ΔRT.

No gender difference was found in RT for the left and right arrow selection task. Some previous studies on gender differences on the CRTT found that men have shorter RTs than women [28, 29], while others, like our study, found no difference between the genders [30]. Dykiert et al. [30] assumed that the CRTT involves both a decision and motor component, and since women are quicker at making decisions [31] and men’s motor performance is faster [32], they cancel each other out, resulting in no gender difference. However, they also admit that this does not explain the different findings in Deary et al. [28] and Der et al. [29]. In these previous studies, participants used their middle and index fingers of both hands and performed a four-choice task using the four fingers. Since we used a different task in our study, a direct comparison is not possible. However, the index fingers of both hands (two fingers) were used in the present study, fewer than those used in previous studies. That said, it could be argued that our task was simpler, which could be one reason we did not identify a gender difference in the arrow task.

There was also no difference between men and women participants in correct response rate on the HMRT, which consistent with Teng and Lee [19] and Seurinck et al. [18], shows that there is no difference in performance in terms of correct responses. However, our findings differed from those of Karadi et al. [20], who found that men had fewer incorrect responses when the hand was shown at a 180° angle. In the hand pictures used in our study, all fingers were extended, and the correct response rate was higher than that in previous studies using various finger positions [9, 20]. Therefore, gender differences may appear when using a complicated hand-shape as the presented image.

Regarding the effect of the hand angle, our findings were consistent with those of previous studies [5, 33, 34]. RT was longer for greater angles, suggesting that participants were using a mental transformation strategy [33] to carry out the task. Furthermore, if a participant wanted to actually rotate his or her own hand to place it over the hand picture, it would be more difficult to rotate the right hand clockwise (45°, 90°, and 135°) and the left hand counter-clockwise (225°, 270°, and 315°). Studies have shown that it takes time to simulate a movement that would take time to actually execute [35]. Informed as such, many previous studies concluded that participants use motor imagery to complete the HMRT [4–9]. Our findings support this, suggesting that both men and women use motor imagery to carry out the task.

ΔRT for the left hand picture was longer for women than for men. Previous HMRT studies found that women’s RT was both longer [20] and shorter [18] than men’s, but neither analyzed the right and left pictures separately. In Karadi et al. [20], it could be assumed that the overall gender difference (for both left and right) resulted because the women’s responses to the pictures of the left hand took longer than the men’s. The gender difference in left–right discrimination could also explain reports of women taking longer to complete tasks that include a transformation of mental rotation component and distinguish between left and right performance [36], and those that indicate that women’s ΔRT is longer than men’s for the left hand picture. Many studies also show that men perform better than do women on the figure MRT [10–16]. Because the HMRT, like the figure MRT, requires participants to perform spatial operations, this difference in performance could explain the gender difference in RT for the picture of the left hand. Butler and colleagues investigated the gender difference in brain activation during a figure MRT [37]. They showed that women performed with a more effortful top-down strategy, because they have higher activity in the dorsal medial prefrontal and other high-order heteromodal association cortices than do men. They also demonstrated that men performed with a more automatic, effortless, and effective bottom-up strategy, because they have higher activity than women in the precuneus and basal ganglia. In this study, male participants may have performed the HMRT using the more efficient bottom-up strategy. Gender differences in the hemispheric lateralization are also assumed one reason for this [38–41]. Stronger lateralization in men than in women to the right hemisphere has been demonstrated during the MRT [38–40, 42], and that to the left hemisphere in a verbal task [41].

Women’s ΔRT for the left hand picture was longer than for the right hand picture. Many previous studies with right-handed participants found their responses to left hand pictures longer than those to right hand pictures [4, 5, 7–9, 43]. This could be because the speed of motor imagery is different for the left and right hands [5, 9, 43]. There is a strong correlation between the speed at which hands are moved in the imagination and at which the motion is actually executed [34]. As such, left hand motor imagery in right-handed participants takes longer than for the right hand in the same way as when the motion is actually executed [44]. Since the speed at which participants in our study mentally rotated their left hands would have been slower than for their right hands, this may have been the reason for the longer RT for the picture of the left hand compared with the picture of the right hand. The late response of the right-handed women to the left hand picture reflects the involvement of handedness in the HMRT. Because it has been reported that the strategy might differ depending on the dominant hand in men [9], a discussion considering handedness may be necessary.

RT and correct response rate have been compared in deciding whether a picture was a left or right hand with amputees who had lost either their dominant or non-dominant hand [45]. The findings of this study, together with those of Ionta and Blanke [5] for RT when participants’ right hands were behind their backs, point to the possibility of participants primarily relying on their dominant hand when using motor imagery to complete a task. Since it is easier to physically rotate and align the left hand with a picture that has been rotated clockwise (45°, 90°, and 135°), participants likely use a mental image of their left hand. If we then assume that they are using a mental image of their right and dominant hand for the other five rotation angles, we can posit the following.

Participants use a mental image of their right hand to align with the hand shown. If they decide that the picture is also of their right hand, they press the key with their right hand. In other words, they do this with the same hand used in the mental rotation. However, if they decide that the picture is of their left hand, they must do this with the hand opposite to the one used in the mental rotation. It has been shown that motor imagery shares some functions with preparing to execute a movement [46]. Since executing a movement with the imagined hand would be faster than executing a movement with the non-imagined hand, RT for a left hand picture rotated clockwise is quicker than for the corresponding right hand, and RT for a right hand picture rotated counter-clockwise (225°, 270°, and 315°) is quicker than for the corresponding left hand. Since right hand motor imagery is used for pictures shown at 0° and 180°, the RT for the picture of the right hand is also shorter than that for the left hand picture. This is why overall, the RT for the right hand pictures was shorter than for the left hand pictures. In addition, if right hand motor imagery is also faster than left hand motor imagery, the difference would increase even further.

There was no difference in men’s ΔRT for left and right hand pictures. While some previous studies on right-handed participants support these findings [33, 47], they are fewer in number than research showing a longer RT for left hand pictures [4, 5, 8, 9, 43]. The present result may suggest that men performed the HMRT using both motor and visual imagery, because the ΔRT for the left hand pictures is likely to be longer if performed by only motor imagery. Seurinck et al. [18] compared brain activity in men and women carrying out an HMRT. They reported that more activity was observed in women’s left ventral premotor cortex, and men had more activity in the lingual gyrus, even though the gender differences were modest. They therefore argued that women may use more motor imagery than do men, and visual or semantic processing could be more important for men. In studies on figure MRT, in which participants form the visual image and mentally rotate it [48], morphologic gender differences have been associated with a performance advantage for men in mental rotation. For example, compared to women, men had a larger anterior hippocampus [17] and greater parietal lobe surface area [49], which are thought to be involved in spatial processing. Wei et al. [17] noted that the role of the anterior hippocampus is to encode information of the head direction and angular features of 3D figures, which are new and abstract for the participants. On the other hand, the hand pictures used in the current study may have not been new and abstract for our participants. However, in the pictures presented at 135°, 180°, and 225°, especially the palm pictures, participants had almost no opportunity to look at their own hands at such angles; therefore, it might be close to new information for them. The participants in the current study watched the presented hand pictures and may have encoded their thumb position or angular features, which are considered to correspond to the head direction in the 3D figure. Therefore, we think it is possible to cite the morphological differences of the anterior hippocampus as a factor influencing gender differences in ΔRT. A greater parietal lobe surface area may also benefit the active manipulation of information [49]. Furthermore, the precuneus is more activated in males than females during figure MRT [37], which is associated with memory in visual imagery [50]. Male participants in the present study might have activated the precuneus during the HMRT and carried out the task using visual imagery.

According to the results of the questionnaire survey, there was no gender difference in performance strategy. Possibly, the motor imagery induced during the HMRT is implicit. Unconsciously induced motor imagery, namely the simulation to superimpose participants’ own hand on the picture, has been suggested in previous studies [4–9, 51, 52]. In other words, as the strategy does not reach consciousness, the estimated RT and results of the questionnaire diverged. The implicit motor imagery induced by HMRT may be related to a mirror neuron system. The premotor cortex and intraparietal sulcus, which are activated during hand movement observation and in which mirror neurons are assumed to be present [53], are similar to the activation area during HMRT [4]. Cheng et al. [54, 55] investigated gender differences in the mirror-neuron system, revealing that women, but not men, displayed stronger activation while observing hand actions other than a moving dot. Indeed, a difference in responses to dominant and non-dominant hand pictures was found in the present study for women, which suggests motor imagery. For men, no significant difference was found in ΔRT between the left and right hand pictures. As such, men might handle hand pictures like objects and perform the HMRT using mainly a visual imagery strategy. Therefore, the gender difference in ΔRT in the present study could also be attributed to the gender difference in the mirror neuron system.

The present study has the limitation of stating neurophysiological differences between men and women based on a low sample size and only reaction times. For future work, we intend to conduct the survey in conjunction with the viewpoint of the ability of visual imagery using figure MRT and with the brain function.

The HMRT is used as a way of potentially inducing motor imagery to relieve pain in complex regional pain syndrome type 1 [56] and to help stroke patients recover the function of upper limb motor impairments [57]. Going forward, to advance the therapeutic uses of HMRT (i.e., therapy using motor imagery), the gender differences demonstrated in this study must be considered. In training to improve paralysis in stroke rehabilitation, the appropriateness of the task difficulty is important. For example, in mental practice training using HMRT for a female patient, an intervention such as lowering the difficulty of the left hand compared to the right hand (reducing the rotation angle) is assumed.

In conclusion, because of the difference in left and right arrow RT, to accurately measure the time taken for the transformation of mental rotation of the hand, it was necessary to subtract the arrow RT from the time taken to press the key after the hand picture was presented and to compare the resulting length of time. For both men and women, ΔRT was longest when the pictures were presented at rotation angles that were difficult to align with their hands. This result suggests that they carried out the HMRT mainly with motor imagery. ΔRT for the left hand picture was found to be longer for women than for men. The women’s ΔRT for the left hand picture was longer than that for the right hand picture, but this difference was not found for men. The strategy for the HMRT is an implicit mental process; therefore, it is difficult to draw a clear conclusion based only on the behavioral experiment. The gender difference might be due to the difference in the balance of motor and visual imageries and the cognitive, neurophysiological, and morphological differences.

## Supporting information

S1 File Original data. (XLSX)
